# Clinical efficacy of free androgen index, a surrogate hallmark of circulating free testosterone level, in male patients with HCV-related chronic liver disease

**DOI:** 10.3164/jcbn.18-30

**Published:** 2018-06-08

**Authors:** Takashi Himoto, Koji Fujita, Teppei Sakamoto, Takako Nomura, Asahiro Morishita, Hirohito Yoneyama, Reiji Haba, Tsutomu Masaki

**Affiliations:** 1 Department of Medical Technology, Kagawa Prefectural University of Health Sciences, 281-1 Hara, Mure-Cho, Takamatsu, Kagawa 761-0123, Japan; 2 Department of Gastroenterology and Neurology, Kagawa University School of Medicine, 1750-1 Ikenobe, Miki-Cho, Kagawa 761-0793, Japan; 3 Department of Diagnosis Pathology, Kagawa University School of Medicine, 1750-1 Ikenobe, Miki-Cho, Kagawa 761-0793, Japan

**Keywords:** free androgen index, HCV, insulin resistance, hepatic fibrosis, hepatic steatosis

## Abstract

The role of free testosterone, that not bound to sex hormone-binding globulin, in male patients with HCV infection remains uncertain. We investigated whether free testosterone is involved in the progression to hepatic fibrosis/steatosis or insulin resistance in male patients with HCV-related chronic liver disease or not. Free androgen indices, which reflect circulating free testosterone levels, were calculated as 100 × total testosterone levels/sex hormone-binding globulin levels in 30 male patients with HCV-related chronic liver disease. Degrees of hepatic fibrosis and steatosis were evaluated by the New Inuyama Classification and the classification proposed by Brunt and colleagues, respectively. Insulin resistance was estimated by HOMA-IR values. Serum total testosterone levels were independent of hepatic fibrosis staging in the enrolled patients. However, circulating sex hormone-binding globulin levels were significantly increased in proportion to the severity of hepatic fibrosis. Therefore, free androgen indices were inversely correlated with the severity of hepatic fibrosis. Moreover, free androgen indices were inversely correlated with the grades of hepatic steatosis and HOMA-IR values in those patients. Our data suggest that lower circulating free testosterone levels may be recognized as the risk factor for more advanced hepatic fibrosis, steatosis and/or higher insulin resistance in male patients with HCV-related chronic liver disease.

## Introduction

Hepatitis C virus (HCV) frequently induces a spectrum of chronic liver diseases from chronic hepatitis to liver cirrhosis, and ultimately to hepatocellular carcinoma (HCC). An estimated more than 184 million people are infected with HCV worldwide at present.^(1)^ It has been well recognized that persistent HCV infection also evokes numerous types of metabolic abnormalities, including insulin resistance, hepatic steatosis, dyslipidemia, and iron overload.^(2–5)^ These metabolic abnormalities, which derive from a variety of host factors and/or the direct action of HCV itself are greatly involved in the development of liver damage and/or fibrosis. Insulin resistance is considered a major cause of hepatic fibrosis and steatosis in patients chronically infected with HCV.^(6)^ We previously revealed that deficiency of trace elements, including zinc and selenium, played pivotal roles in exacerbation of insulin resistance in patients with HCV-related chronic liver disease (CLD-C).^(7,8)^

On the other hand, males appear to be more at risk for advanced liver diseases, including liver cirrhosis and HCC.^(9)^ Indeed, sex hormones such as testosterone and estradiol are likely to affect the progression to hepatic fibrosis and insulin resistance in patients with chronic liver diseases. White and colleagues previously elucidated that higher serum testosterone levels might result in more advanced hepatic fibrosis in male patients with CLD-C.^(10)^ Other studies have shown that lower serum total testosterone concentrations were associated with more advanced hepatic fibrosis,^(11)^ or that serum total testosterone levels were unrelated to the degree of hepatic fibrosis in those patients.^(12)^ Clearly, the involvement of testosterone in the progression to hepatic fibrosis remains controversial.

Testosterone also plays a crucial role in lipid homeostasis.^(13)^ A decrease in the level of circulating total testosterone was observed in sera of patients with nonalcoholic fatty liver disease (NAFLD) in inverse proportion to the grade of hepatic steatosis.^(14)^ In addition, testosterone also has a protective action on pancreatic beta cells.^(15)^ Consequently, a lower level of testosterone eventually causes higher insulin resistance in patients with type 2 diabetes mellitus (T2DM) or metabolic syndrome.^(16,17)^ A closely inverse relationship exists between serum testosterone level and degree of obesity in men.^(18)^

In female CLD-C patients, menopausal status has been associated with more advanced hepatic fibrosis.^(19)^ By contrast, menopausal CLD-C patients who received a hormone replacement therapy showed milder-stage hepatic fibrosis than those who did not,^(20)^ implying that estrogen has a protective effect against the progression to severe hepatic fibrosis in female patients with CLD-C. Menopause has also been suggested to accelerate the development of hepatic fibrosis in patients with nonalcoholic steatohepatitis (NASH).^(21)^

Testosterone is a major androgen, secreted almost exclusively by the testes. Testosterone circulates in three forms: bound to sex hormone-binding globulin (SHBG), bound to albumin or other plasma proteins, and a free (unbound) state^(22)^ Around 50–60% of total testosterone tightly binds to SHBG, and 40–50% of total testosterone is loosely bound to albumin. Therefore, unbound-type testosterone, termed “free testosterone”, comprises only 1–2% of total testosterone. Free testosterone and albumin-bound testosterone has been considered to represent the active testosterone state. However, the fraction may vary in the target tissues and pathophysiological conditions. Hence, a reliable biochemical marker of androgen activity is missing.

SHBG is a 90 kDa glycoprotein composed of two 373-amino acid subunits, and is largely synthesized in the liver.^(23)^ SHBG plays a crucial role in modulating the biological activities of sex hormones as well as transporting sex hormones to target tissues. Previous studies documented that a lower levels of serum SHBG was significantly associated with insulin resistance in patients with T2DM or metabolic syndrome.^(24,25)^ By contrast, circulating SHBG concentrations in sera of patients with liver cirrhosis were significantly higher compared to those in the sera of normal healthy controls.^(26)^

The primary purpose of the present study was to investigate the correlation between circulating total or free testosterone concentrations and insulin resistance in male patents with CLD-C. Moreover, we sought to verify whether serum total or free testosterone levels were associated with hepatic fibrosis or steatosis in those patients.

## Materials and Methods

### Study population

Thirty male patients with CLD-C were randomly selected from patients admitted to the Hospital of Kagawa University School of Medicine between 2003 and 2015. All of the selected CLD-C patients had detectable serum HCV RNA as determined by polymerase chain reaction (PCR) and showed histological findings compatible with chronic hepatitis or liver cirrhosis.

The study protocol complied with all of the provisions of the Declaration of Helsinki. The design of this study was approved by the Ethical Committee of the Kagawa University School of Medicine, and informed consent was obtained from each individual before liver biopsy.

### Laboratory assessments

Fasting blood samples were taken in the morning for measurements of plasma glucose and serum immunoreactive insulin (IRI), alanine aminotransferase (ALT), total cholesterol (T-Cho), triglyceride (TG), total testosterone, and SHBG. Plasma glucose and serum IRI, ALT, T-Cho and TG levels were measured using standard laboratory techniques. Serum total testosterone levels were determined by an electro chemiluminescence immunoassay. Serum SHBG levels were assessed by a commercially available enzyme-linked immunosorbent assay (ELISA) kit (Bio Vendor Laboratory Medicine Inc, Palackeho, Czech Republic). Serum free testosterone levels are difficult to measure directly. Instead, free androgen index (FAI) was designated as a surrogate of the circulating free testosterone level using the following equation:^(27)^ FAI = 100 × total testosterone/SHBG. Insulin resistance was evaluated based on the homeostasis model for the assessment of insulin resistance (HOMA-IR) value using following equation: HOMA-IR value = fasting insulin (µU/ml) × fasting glucose (mg/dl)/405. Insulin resistance was defined as a HOMA-IR value exceeding 2.5. Body mass index (BMI) was estimated as a hallmark of obesity. Obesity was defined as a BMI over 25.0 kg/m^2^, because the proportion of the Japanese population with a BMI higher than 30 kg/m^2^ has been reported to be less than 2–3%,^(28)^ while the proportion of obesity in Western countries ranges from 10 to 20%.^(29)^ Quantitative detection of serum HCV RNA was performed using the Amplicor-HCV monitor assay (Roche Diagnostics, Tokyo, Japan)^(30)^ or COBAS TaqMan HCV test (Rosche Diagnostics).^(31)^ The values measured by the Amplicor-HCV monitor assay were adjusted to those determined by the TaqMan assay. The HCV genotypes were determined by the HCV RNA genotyping assay system.^(32)^

These biochemical and immunological data were obtained from the enrolled patients before liver biopsy. These parameters in the study subjects were determined before any treatment.

### Histological assessments

Liver tissue specimens were obtained by liver biopsy under ultrasound guidance, using 16-gauge needles, before treatments. The tissue samples were fixed in 10% formalin, embedded in paraffin, and then sectioned. The tissue sections were stained with hematoxylin and eosin for morphological evaluation. The degrees of hepatic fibrosis (staging) were evaluated, using the New Inuyama Classification, a standard criterion for histological assessment of chronic hepatitis in Japan.^(33)^ In brief, the stages of hepatic fibrosis were divided into F_0 _to F_4_. F_0_ was defines as no fibrosis in the tissue specimens, while F_4_ was defined as liver cirrhosis. Grades of hepatic steatosis were assigned using the classification proposed by Brunt and colleagues.^(34)^ Hepatic steatosis observed in none, less than 33%, 33–66%, or more than 66% of hepatocytes was designated as grade 0, 1, 2, or 3, respectively.

### Statistical analyses

Data values are represented as means ± SD. The Bonferroni/Dunn method were applied for comparisons of three or more groups. The relationships among quantitative variables were analyzed by Pearson’s test. *P* values less than 0.05 were considered significant.

## Results

### Patients characteristics

The clinical characteristics of the enrolled CLD-C patients in this study are shown in Table 1. In terms of fibrosis degree, nine of 30 (30%) CLD-C patients were F_1_, eight (27%) were F_2_, nine (30%) were F_3_, while four of 30 (13%) CLD-C patients showed liver cirrhosis (F_4_). On the other hand, fifteen of 30 (50%) CLD-C patients had no steatosis (grade 0), while nine (30%) and six (20%) CLD-C patients had grade 1 and grade 2 steatosis, respectively. None of the CLD-C patients had grade 3 steatosis. Nine of the 30 (30%) CLD-C patients fulfilled the category for insulin resistance. Thirteen of the 30 (43%) CLD-C patients were in the category for obesity in Japan.

### Correlation between serum total testosterone and SHBG levels in the enrolled male patients

**Fig. 1:**
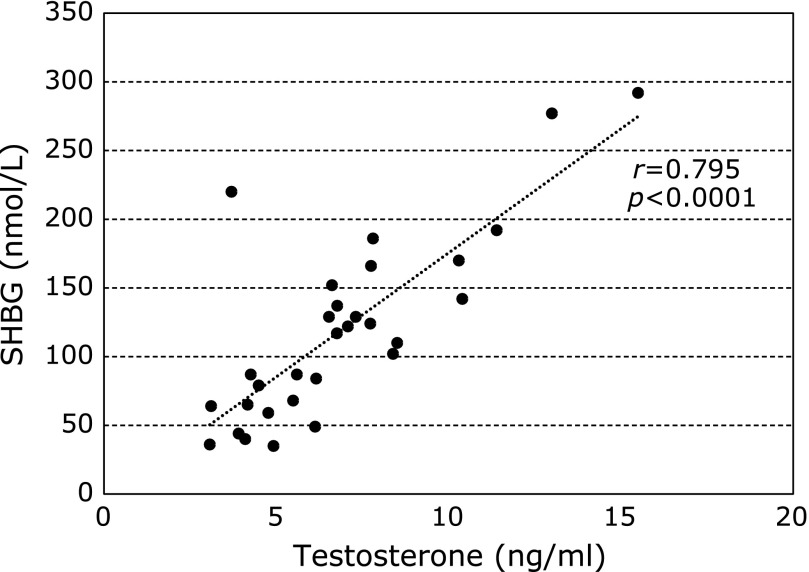
Relationship between serum total testosterone and SHBG levels in male patients with CLD-C. CLD-C, HCV-related chronic liver disease; SHBG, sex hormone-binding globulin.

We investigated the relationship between serum total testosterone and SHBG levels in the enrolled male CLD-C patients. As shown in Fig. 1, a close correlation was found between serum total testosterone and SHBG levels in those patients (*r* = 0.795, *p*<0.0001), supporting that circulating SHBG concentrations may depend on total testosterone concentrations.

### Correlations between the severity of hepatic fibrosis and serum total or free testosterone level

**Fig. 2:**
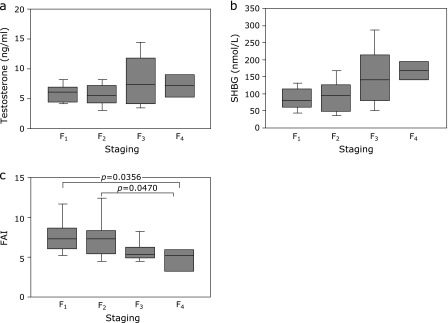
Relationships between the degrees of hepatic fibrosis and (a) total testosterone levels, (b) SHBG levels or (c) FAIs in male CLD-C patients. The boxes represent the values within 25th and 75th percentiles. The horizontal bars represent the medians. CLD-C, HCV-related chronic liver disease; FAI, free androgen index; SHBG, sex hormone-binding globulin.

The association of the staging degree with serum total testosterone concentration was examined in patients with CLD-C. Figure 2a shows no significant differences in serum total testosterone levels between the groups of F_1_ through F_4_. In contrast, circulating SHBG levels were increased in proportion to the severity of hepatic fibrosis (F_1_, 84.0 ± 33.3 nmol/L; F_2_, 94.5 ± 51.6 nmol/L; F_3_, 153.2 ± 87.4 nmol/L; F_4_, 168.3 ± 42.1 nmol/L; Fig. 2b). Accordingly, FAIs, the surrogate hallmark of free testosterone levels, were significantly lower in the group of F_4_ than that in the groups of F_1_ or F_2_ (4.58 ± 2.10 vs 7.68 ± 2.40, *p* = 0.0356, 4.58 ± 2.10 vs 7.55 ± 3.07, *p* = 0.0470, Fig. 2c).

### Correlation between hepatic steatosis and serum testosterone or SHBG level

**Fig. 3:**
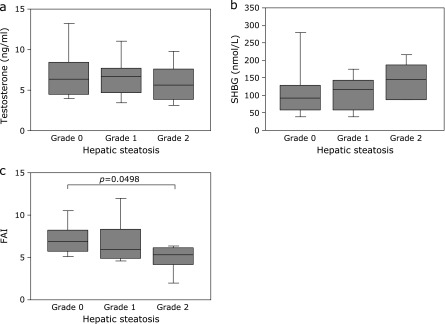
Relationships between the grade of hepatic steatosis and (a) total testosterone levels, (b) SHBG levels or (c) FAIs in male CLD-C patients. The boxes represent the values within 25th and 75th percentiles. The horizontal bars represent the medians. CLD-C, HCV-related chronic liver disease; FAI, free androgen index; SHBG, sex hormone-binding globulin.

Next, we investigated whether the grade of hepatic steatosis was correlated with serum total or free testosterone levels in male patients with HCV-related CLD. Serum total testosterone concentrations were decreased in proportion to the grade of hepatic steatosis in male patients with CLD-C (Fig. 3a). Circulating SHBG levels in patients with grade 2 steatosis tended to be higher than those in patients with grade 0 or grade 1 steatosis (Fig. 3b). Accordingly, patients with grade 2 steatosis had significantly lower FAIs than those with grade 0 steatosis (4.85 ± 1.74 vs 7.26 ± 2.18, *p* = 0.0498, Fig. 3c).

### Correlation between insulin resistance and serum testosterone or SHBG level

**Fig. 4:**
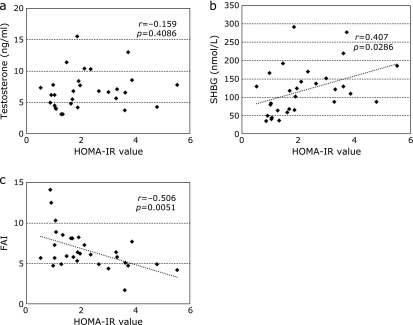
Relationships between HOMA-IR values and (a) total testosterone levels, (b) SHBG levels or (c) FAIs in male CLD-C patients. CLD-C, HCV-related chronic liver disease; FAI, free androgen index; HOMA-IR, homeostasis model for assessment of insulin resistance; SHBG, sex hormone-binding globulin.

Similarly, the association of insulin resistance with serum total or free testosterone levels was analyzed in the enrolled patients. Serum total testosterone levels were not correlated with the HOMA-IR values (*r* = −0.159, *p* = 0.4086, Fig. 4a), while the circulating SHBG level was significantly related to the HOMA-IR values (*r* = 0.407, *p* = 0.0286, Fig. 4b). Hence, FAIs were inversely correlated with the HOMA-IR values (*r* = −0.506, *p* = 0.0051, Fig. 4c).

### Other host factors associated with FAIs

**Fig. 5:**
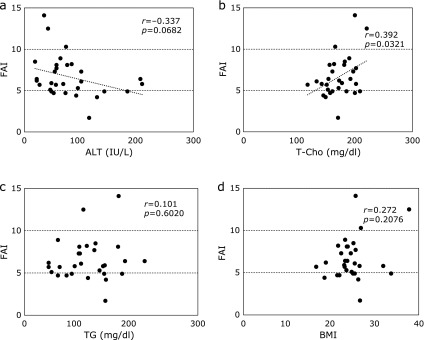
Relationships between FAIs and (a) serum ALT levels, (b) serum T-Cho levels, (c) serum TG levels or (d) BMIs in male CLD-C patients. ALT, alanine aminotransferase; BMI, body mass index; CLD-C, HCV-related chronic liver disease; FAI, free androgen index; T-Cho, total cholesterol; TG, triglyceride.

We also investigated whether other host factors, including serum ALT, T-Cho and TG levels and BMI, were associated with total or free testosterone levels among male CLD-C patients. No significant correlation was found between serum total testosterone and ALT levels in male CLD-C patients. However, FAIs tended to be reduced in inverse proportion to serum ALT levels in the enrolled patients (*r* = −0.337, *p* = 0.0682, Fig. 5a). FAIs were roughly associated with serum T-Cho levels in those patients (*r* = 0.392, *p* = 0.0321, Fig. 5b), but not with serum TG levels (*r* = 0.101, *p* = 0.6020, Fig. 5c). FAIs were independent of BMIs (*r* = 0.272, *p* = 0.2076, Fig. 5d), suggesting that free testosterone was not involved in the process of obesity in male CLD-C patients.

### Correlation between FAIs and loads of HCV RNA or HCV genotypes

**Fig. 6:**
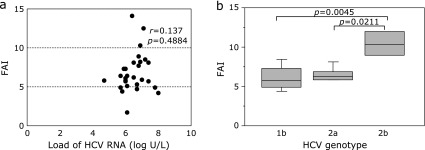
Relationships between FAIs and (a) loads of HCV RNA or (b) HCV genotypes. The boxes represent the values within 25th and 75th percentiles. The horizontal bars represent the medians.

Association of viral factors, including loads of HCV-RNA and HCV genotypes, with testosterone status was investigated in male CLD-C patients. No significant correlation was observed between loads of HCV RNA and FAIs in the enrolled patients (*r* = 0.137, *p* = 0.4884, Fig. 6a). However, mean FAIs were significantly higher in the group of HCV genotype 2b than those in the groups of genotype 1b or genotype 2a [10.43 ± 2.00 vs 6.19 ± 2.41, *p* = 0.0045 (genotype 1b) or 6.46 ± 0.95, *p* = 0.0211 (genotype 2a); Fig. 6b].

### Correlations between insulin resistance and hepatic fibrosis or steatosis

**Fig. 7:**
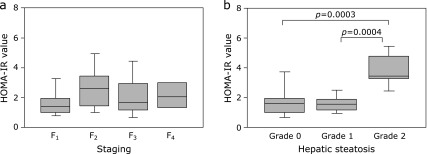
Relationships between HOMA-IR values and the degrees of (a) hepatic fibrosis or (b) hepatic steatosis in male patients with CLD-C. The boxes represent the values within 25th and 75th percentiles. The horizontal bars represent the medians. CLD-C, HCV-related chronic liver disease; HOMA-IR, homeostasis model for assessment of insulin resistance.

Figure 7a shows the HOMA-IR value in each staging of hepatic fibrosis. There were no significant differences in the values of HOMA-IR among the four stages, indicating that hepatic fibrosis was independent of insulin resistance in the enrolled CLD-C patients. However, CLD-C patients with grade 2 steatosis had significantly higher HOMA-IR values than those with grade 0 or 1 steatosis [3.82 ± 1.15 vs 1.80 ± 1.10, *p* = 0.0003 (grade 0) or 1.61 ± 0.58, *p* = 0.0004, (grade 1); Fig. 7b], implying that hepatic steatosis may depend on insulin resistance in CLD-C patients.

## Discussion

Our present data elucidated that FAIs were inversely correlated with HOMA-IR values in male patients with CLD-C, supporting the notion that circulating lower free testosterone levels might result in the exacerbation of insulin resistance in those patients. Some previous studies have demonstrated a relationship between a decrease in the serum total/free testosterone level and the exacerbation of insulin resistance in patients with type 2 DM or metabolic syndrome.^(14,17)^ However, to the best of our knowledge, this is the first report to reveal an inverse correlation between circulating free testosterone levels and insulin resistance in male CLD-C patients.

The mechanism by which circulating lower free testosterone levels may evoke the exacerbation of insulin resistance in such patients remains uncertain. A putative explanation is that an accumulation of visceral adipose tissue (VAT), which exacerbates insulin resistance in male patients, is itself associated with lower free testosterone levels.^(35)^ Lower free testosterone levels are likely to increase the risk of VAT accumulation, which results in liver exposure to higher free fatty acid levels and potentially evokes higher insulin resistance in male CLD-C patients. Another explanation for the association of lower free testosterone concentration with higher insulin resistance may be that the impaired function of Leydig cells directly leads to hyperinsulinemia among those patients.^(36)^

We showed that circulating total testosterone levels were decreased, while serum SHBG levels were increased in proportion to the grade of hepatic steatosis among male CLD-C patients. Therefore, free testosterone levels were significantly decreased in grade 2 steatosis, compared to those in grade 0 steatosis. Lower free testosterone levels as well as lower total testosterone levels were previously observed in male NAFLD patients.^(37)^ This result supports the hypothesis that a decline in free testosterone may cause the progression to hepatic steatosis in male CLD-C patients as well.

One of the plausible mechanisms underlying the correlation between lower free testosterone and more advanced hepatic steatosis among male CLD-C patients is shown as follows: a lower serum free testosterone level leads to the accumulation of VAT, which can subsequently initiate exposure of the liver to higher amounts of free fatty acid. The present study confirmed that the severity of hepatic steatosis was significantly associated with insulin resistance in male CLD-C patients. Therefore, higher insulin resistance may result in the exacerbation of hepatic steatosis among those patients.

Another explanation for the mechanism is that testosterone may inhibit the activity of lipoprotein lipase, which reduces the uptake of TG by adipocytes in men.^(38)^ A decline in testosterone may substantially augment the activity of hormone-sensitive lipase, and consequently accumulate fat deposition in the liver.

Taking these results described above into consideration, testosterone replacement therapy may improve visceral adipocity and insulin resistance in male CLD-C patients. Indeed, there is some evidence that testosterone replacement therapy improves visceral adipocity and insulin resistance in hypogonadal male patients with type 2 diabetes mellitus.^(39)^

Our results elucidated that serum free testosterone levels were decreased in inverse proportion to the severity of hepatic fibrosis among male CLD-C patients. The elevation of SHBG levels with no alteration in total testosterone levels may account for calculated lower free testosterone levels in CLD-C patients with advanced hepatic fibrosis. Estradiol is likely to play a crucial role in the augmentation of SHBG synthesis in the liver.^(40)^ Previous studies have confirmed that free testosterone levels were reduced in patients with advanced hepatic fibrosis,^(11,12)^ although the influence of total testosterone remains controversial in male patients infected with HCV. Therefore, free testosterone levels should be determined in cirrhotic patients.^(41)^ In the present study, the severity of hepatic fibrosis was not associated with insulin resistance among male CLD-C patients. Further examinations will be required to clarify the mechanisms by which lower free testosterone results in more severe hepatic fibrosis in such patients.

Testosterone status affects various components of the lipid profile.^(13,42)^ To our surprise, however, this study failed to demonstrate a relationships between serum TG and free testosterone levels in male CLD-C patients, despite the inverse correlation found between free testosterone levels and grades of hepatic steatosis. Circulating free testosterone levels were associated with T-Cho levels, but total testosterone levels were not (data not shown in the text). Unfortunately, there are not enough data to explain the reasons for this discrepancy in the present study.

With regard to the relationship between testosterone and hepatic inflammation, White and colleagues demonstrated a positive correlation between total testosterone level and hepatic inflammation as well as hepatic fibrosis in male patients infected with HCV.^(10)^ In patients with NAFLD and T2DM, serum SHBG levels were inversely associated with serum ALT levels.^(43)^ Our results showed that the concentration of free testosterone, but not total testosterone, tended to be decreased as the grade of hepatic inflammation developed in male CLD-C patients. A recent study demonstrated that male cholangitis-model mice could be rendered susceptible to liver inflammation by castration, indicating that testosterone deficiency resulted in hepatic inflammation in this mouse model.^(44)^

A previous study showed that the HCV core protein augmented androgen receptor signaling in experimental animals.^(45)^ In this study, however, HCV itself may not be involved in the alteration of free testosterone levels in male CLD-C patients, because free testosterone levels were unrelated to the load of HCV-RNA. Free testosterone levels in genotype 2b were higher than those in other genotypes. Unfortunately, the group of HCV genotype 2b was a particularly small-sized sample (*n* = 3). A larger scale cohort study will be needed to confirm whether free testosterone levels depend on HCV genotype or not.

There were several limitations worth mentioning in the present study. First, our study was limited by small sample size, although many useful results were obtained. Therefore, a larger-scale cohort study is required for confirmation of results. Second, we did not adjust serum testosterone levels in the enrolled patients. Serum testosterone levels are gradually decreased in association with aging.^(46)^ However, the ages in 24 of our 30 patients (80%) were greater than 50 years old (data not shown in the text). Therefore, it is unlikely that aging affects serum testosterone levels in this study. Third, we did not directly determine free testosterone levels, but used FAIs as its surrogate in male CLD-C patients. However, an extremely close correlation was previously confirmed between free testosterone levels and FAIs in patients with liver cirrhosis.^(26)^ FAI seems to be widely recognized as a surrogate marker reflecting serum free testosterone level.

In conclusion, our results suggest that a decline in circulating free testosterone concentration might lead to more advanced hepatic fibrosis and/or steatosis, and higher insulin resistance in male CLD-C patients. Insulin resistance was associated with insulin resistance, but not hepatic fibrosis in the enrolled patients.

## Figures and Tables

**Table 1:** Clinical characteristics of the enrolled male patients with CLD-C

Age (years)	56.0 ± 10.3 (31–70)
BMI	24.6 ± 4.2 (17.0–31.9)
Staging (F1/F2/F3/F4)	9/8/9/4
Steatosis (grade 0/1/2)	15/9/6
HCV genotype (1b/2a/2b)	22/5/3
Loads of HCV-RNA (log IU/ml)	6.52 ± 0.71 (4.70–8.00)
Values of HOMA-IR	2.12 ± 1.29 (0.52–5.53)

## Abbreviations

ALT: alanine aminotransferase
BMI: body mass index
CLD-C: HCV-related chronic liver disease
FAI: free androgen index
HCC: hepatocellular carcinoma
HCV: hepatitis C virus
HOMA-IR: homeostasis model for assessment of insulin resistance
NASH: nonalcoholic steatohepatitis
SHBG: sex hormone-binding globulin
T-Cho: total cholesterol
TG: triglyceride
T2DM: type 2 diabetes mellitus
VAT: visceral adipose tissue
